# Bioinformatics strategies to identify differences in molecular biomarkers for ischemic stroke and myocardial infarction

**DOI:** 10.1097/MD.0000000000035919

**Published:** 2023-11-17

**Authors:** Min Wang, Yuan Gao, Huaqiu Chen, Ying Shen, Jianjie Cheng, Guangming Wang

**Affiliations:** a School of Clinical Medicine, Dali University, Dali, Yunnan, P.R. China; b School of Clinical Medicine, Zhengzhou University, Zhengzhou, Henan, P.R. China; c Xichang People’s Hospital, Xichang, Sichuan, P.R. China; d The First Hospital of Liangshan, Xichang, Sichuan, P.R. China; e The First Affiliated Hospital of Dali University, Yunnan, P.R. China; f Center of Genetic Testing, The First Affiliated Hospital of Dali University, Dali, Yunnan, P.R. China.

**Keywords:** bioinformatics, biomarker, ischemic stroke, myocardial infarction

## Abstract

Ischemic strokes (ISs) are commonly treated by intravenous thrombolysis using a recombinant tissue plasminogen activator; however, successful treatment can only occur within 3 hours after the stroke. Therefore, it is crucial to determine the causes and underlying molecular mechanisms, identify molecular biomarkers for early diagnosis, and develop precise preventive treatments for strokes. We aimed to clarify the differences in gene expression, molecular mechanisms, and drug prediction approaches between IS and myocardial infarction (MI) using comprehensive bioinformatics analysis. The pathogenesis of these diseases was explored to provide directions for future clinical research. The IS (GSE58294 and GSE16561) and MI (GSE60993 and GSE141512) datasets were downloaded from the Gene Expression Omnibus database. IS and MI transcriptome data were analyzed using bioinformatics methods, and the differentially expressed genes (DEGs) were screened. A protein–protein interaction network was constructed using the STRING database and visualized using Cytoscape, and the candidate genes with high confidence scores were identified using Degree, MCC, EPC, and DMNC in the cytoHubba plug-in. Gene Ontology (GO) and Kyoto Encyclopedia of Genes and Genomes (KEGG) pathway enrichment analyses of the DEGs were performed using the database annotation, visualization, and integrated discovery database. Network Analyst 3.0 was used to construct transcription factor (TF) – gene and microRNA (miRNA) – gene regulatory networks of the identified candidate genes. The DrugBank 5.0 database was used to identify gene–drug interactions. After bioinformatics analysis of IS and MI microarray data, 115 and 44 DEGS were obtained in IS and MI, respectively. Moreover, 8 hub genes, 2 miRNAs, and 3 TFs for IS and 8 hub genes, 13 miRNAs, and 2 TFs for MI were screened. The molecular pathology between IS and MI presented differences in terms of GO and KEGG enrichment pathways, TFs, miRNAs, and drugs. These findings provide possible directions for the diagnosis of IS and MI in the future.

## 1. Introduction

Globally, stroke is characterized by high morbidity and mortality and is the second leading cause of death.^[1]^ The prevalence of stroke has increased by 20.5% since 2012, and it is predicted that by 2030, approximately 3.9% of adults in the United States will have had a stroke.^[2]^ Ischemic stroke (IS) accounts for 87% of all strokes.^[3]^ Moreover, acute IS usually occurs suddenly without any distinct early warning sign. It progresses rapidly and can lead to serious complications and even long-term disability.^[1]^ The most common treatment for IS is intravenous thrombolysis using recombinant tissue plasminogen activator; however, the window for treatment is only 3 hours.^[4]^ Thus, it is crucial to determine the causes and underlying molecular mechanisms of stroke, identify molecular biomarkers for early diagnosis, and develop precise preventive treatments.^[5]^

Myocardial infarction (MI) is caused by the blockage of blood flow in one of the main coronary arteries supplying the heart muscle, usually because of the rupture of atherosclerotic plaques and thrombosis. Prolonged ischemia leads to expansion, necrosis, apoptosis, and necrotic death of the myocardium.^[6]^ MI is a common disease with a high mortality rate that results in health and economic challenges worldwide.^[7]^ Furthermore, acute MI (AMI) is the leading cause of disability and death worldwide.^[8]^ Cardiogenic shock after MI is most commonly caused by severe left ventricular dysfunction and is the most serious complication of AMI, leading to mortality and morbidity.^[9]^ Furthermore, treatment options for heart failure after MI are limited.^[10]^ IS is a serious complication after the onset of MI.^[8]^ Thus, new biomarkers should be identified to better diagnose and treat MI. Although there is evidence of the pathophysiological relationship between IS and MI, to the best of our knowledge, differences between the molecular markers of the 2 diseases have not been reported. Therefore, investigating the differences between the 2 diseases at the molecular level will help better analyze their respective pathogenesis and provide a more accurate direction for future diagnosis and treatment.

In this study, we aimed to distinguish the molecular markers of IS and MI using bioinformatics methods and to provide possible directions for the diagnosis of IS and MI in the future.

## 2. Materials and methods

As all the data were obtained from online databases, written informed consent from patients had already been obtained. Moreover, our study was based on open-source data; thus, there are no relevant ethical issues.

### 2.1. Data collection

**Figure 1.**
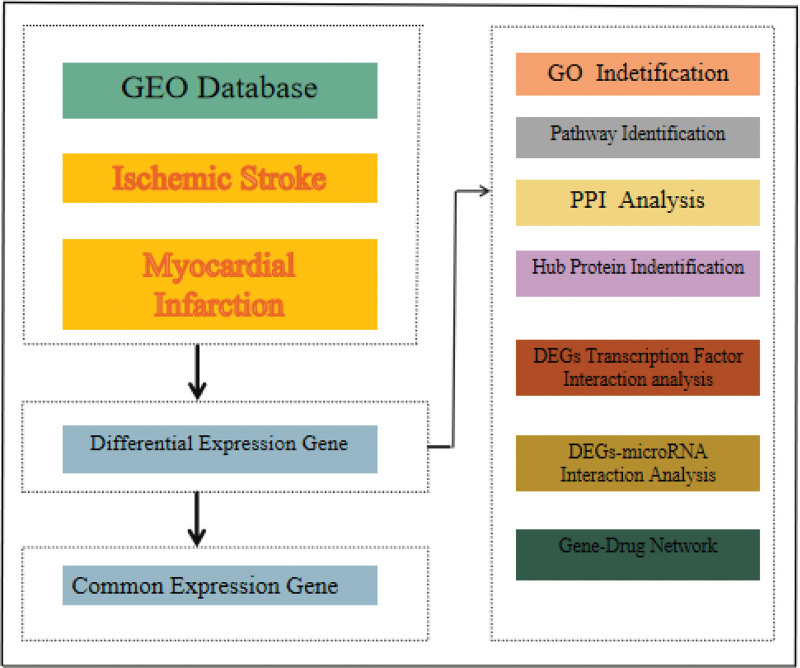
Workflow chart. DEGs = differentially expressed genes, GO = Gene Ontology, PPI = protein–protein interaction.

Figure 1 shows the workflow followed during this study. The keywords “myocardial infarction,” “ischemic stroke,” and “*Homo sapiens*” were searched in the National Center for Biotechnology Information Gene Expression Omnibus (GEO) database (https://www.ncbi.nlm.nih.gov/geo/). After screening, 4 datasets (GSE58294, GSE16561, GSE60993, and GSE141512) were identified as target datasets, and the corresponding gene expression files were downloaded.

The GSE58294 dataset was identified from the GPL570 sequencing platform and included 69 cardioembolic stroke and 23 control group samples. The GSE16561 dataset was identified from the GPL6883 sequencing platform and included 39 patients with IS and 24 healthy control samples. The GSE60993 dataset was identified from the GPL6884 sequencing platform and included 17 patients with MI and 16 healthy cohort participants. The GSE141512 dataset was identified from the GPL17586 sequencing platform and included 6 patients with MI and 6 healthy cohort participants. The gene expression matrix and associated annotation files for each array dataset were downloaded, and the probe IDs were converted to gene names using the corresponding annotation files. Multiple probes were averaged for mapping to the same symbol. Using perl v5.30.0 software, the 2 IS datasets, GSE58294 and GSE116561, and the 2 MI datasets, GSE60993 and GSE141512, were combined to extract the same genes.

### 2.2. Identification of differentially expressed genes (DEGs)

**Figure 2.**
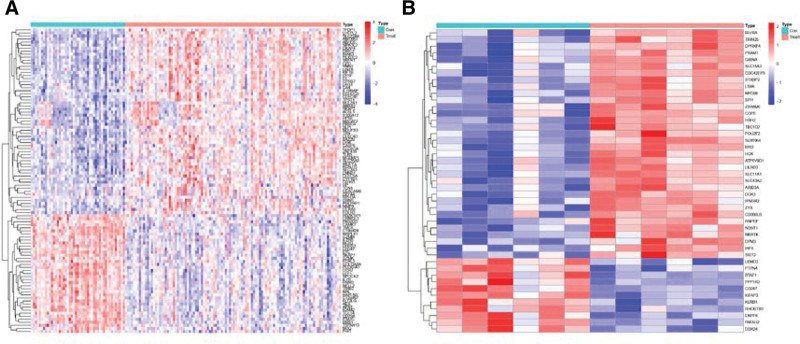
Heatmaps of differentially expressed genes (DEGs) in ischemic stroke (IS) and myocardial infarction (MI). (A) Heatmap of DEGs in IS. (B) Heatmap of DEGs in MI.

**Figure 3.**
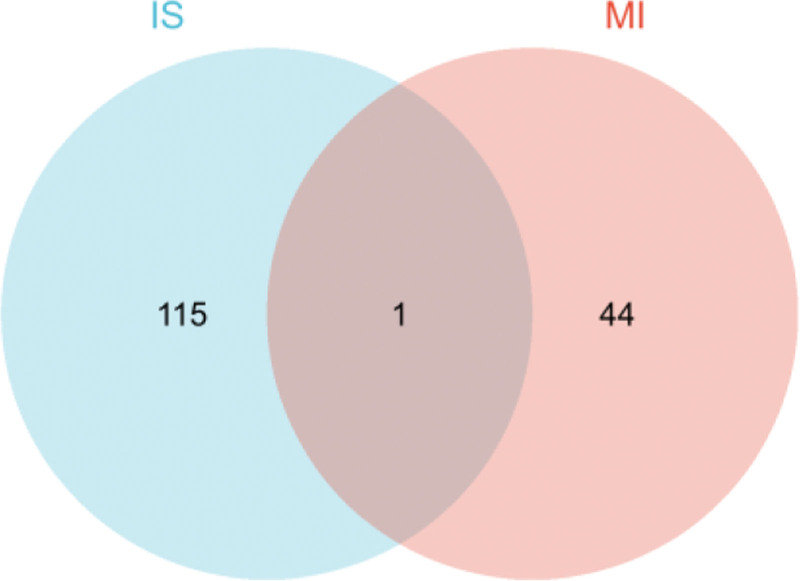
Venn diagram showing common differentially expressed genes (DEGs) in ischemic stroke (IS) and myocardial infarction (MI).

The respective microarray datasets of the 2 diseases were integrated to generate one large dataset via the surrogate variable analysis package in R (R Foundation for Statistical Computing, Vienna, Austria), which is widely used to remove batch effects in high-throughput experiments. The “limma” and “pheatmap” R packages were used to filter genes with missing values and vacancies according to the screening conditions, |logFC| > 0.58 and adj.P.Val.Filter ≤ 0.05. Several important genes were identified for each disease, and the differential dysregulation of the identified genes was used to predict the occurrence of the disease.^[11]^ DEG heatmaps were generated (Fig. 2). A Venn diagram was used to represent the key differential genes common among the 4 datasets (Fig. 3).

### 2.3. Enrichment analysis for significant Gene Ontology (GO) and molecular pathway selection

To explore the cellular pathways and GO characteristics affected by DEGs, the database for annotation, visualization, and integrated discovery software Metascape (https://metascape.org/gp/index.html#/main/step1) was used to perform Kyoto Encyclopedia of Genes and Genomes (KEGG) pathway and GO enrichment analyses on key DEGs using the following criteria: min overlap, 3; *P* value cutoff, .01; and min enrichment, 3.

### 2.4. Analysis of protein–protein interactions (PPIs)

The PPI network is at the core of the cellular/molecular mechanism that constitutes the interaction between 2 or more protein components.^[12]^ Here, the online STRING database (https://string-db.org/) was used to construct PPI networks of proteins encoded by the selected genes using Cytoscape v3.9.1. The cutoff criterion was a confidence score ≥ 0.4. Candidate gene-encoded protein interactions were analyzed.

### 2.5. Analysis of transcription factors (TFs) and miRNAs

NetworkAnalyst (https://www.networkanalyst.ca/) was used to visualize the interaction network between DEG-TFs and DEG-miRNAs. JASPAR (https://jaspar.genereg.net/) and the Encyclopedia of DNA Elements (ENCODE) databases were used to identify and study DEG-TF relationships. The TarBase v8.0 and miRTarBase v8.0 databases were used to study miRNA-DEG interactions.

### 2.6. Drug–gene interaction database

NetworkAnalyst was used to identify possible drugs to treat IS, MI, and associated diseases. The drug predictions were performed using the DrugBank 5.0 database (https://go.drugbank.com/).

## 3. Results

### 3.1. Identification results of DEG analyses

A total of 115 IS and 44 MI DEGs as well as one common DEG were screened. Among the 115 IS DEGs, the expressions of 76 and 39 were upregulated and downregulated, respectively. Among the 44 MI DEGs, the expressions of 33 and 11 were upregulated and downregulated, respectively.

### 3.2. KEGG and GO enrichment analyses

**Figure 4.**
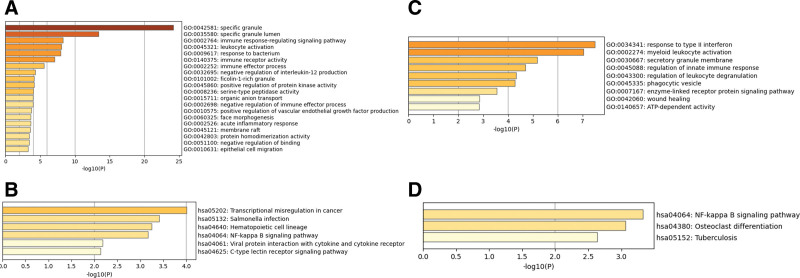
Results of Gene Ontology (GO) and Kyoto Encyclopedia of Genes and Genomes (KEGG) pathway enrichment analyses of differentially expressed genes (DEGs). (A) Results of GO enrichment analysis for ischemic stroke (IS). (B) Results of KEGG enrichment analysis for IS. (C) Results of GO enrichment analysis for myocardial infarction (MI). (D) Results of KEGG enrichment analysis for MI.

Highly expressed GO terms were used to identify disease-related molecules. Functional GO terms were divided into 3 categories: molecular function, biological process, and cellular component. The enrichment and classification results of the important IS and MI pathways were selected and are displayed in Figure 4.

### 3.3. Construction and analysis of PPI network

**Figure 5.**
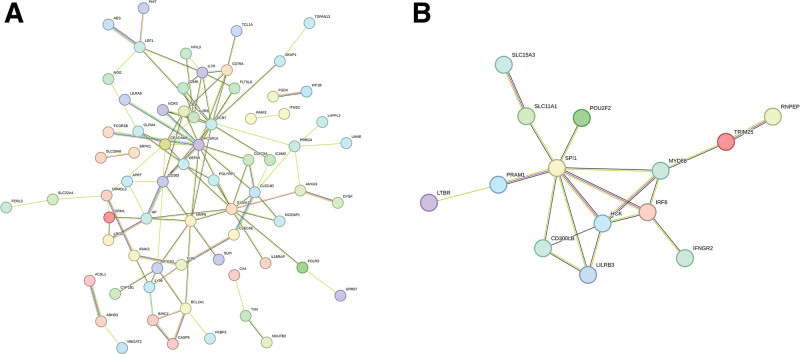
Protein–protein interaction (PPI) network diagram. (A) Ischemic stroke PPI network. (B) Myocardial infarction PPI network. Proteins are represented by colored circles, and connections between proteins are characterized by edges.

**Figure 6.**
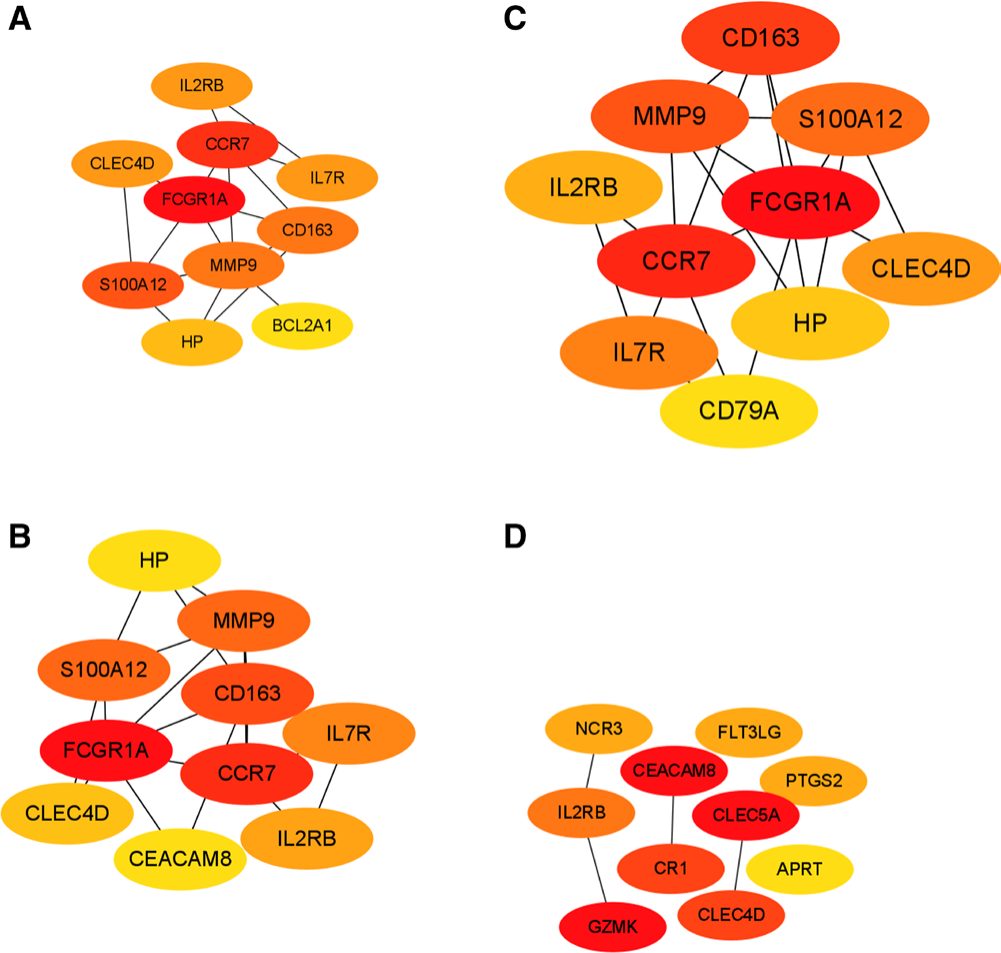
Hub proteins in ischemic stroke identified using 4 different cytoHubba algorithms. (A) Degree, (B) MCC, (C) EPC, (D) DMNC.

**Figure 7.**
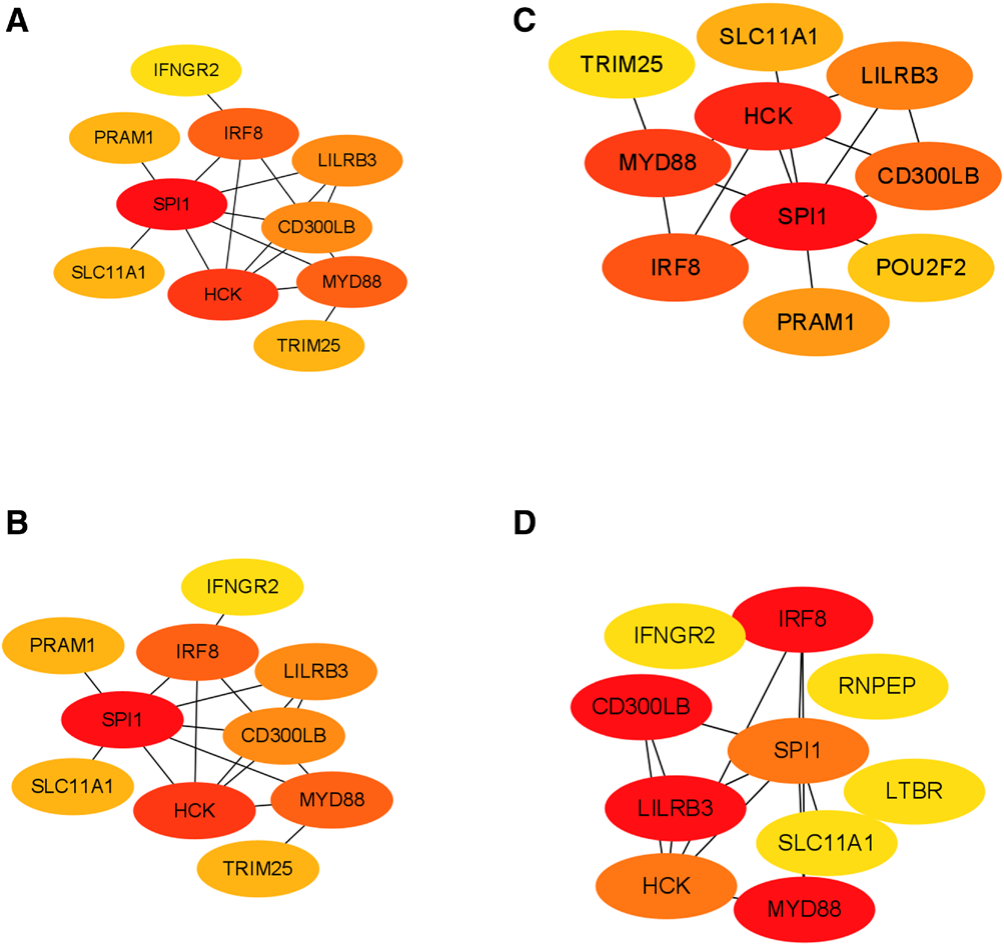
Hub proteins of myocardial infarction identified using 4 different cytoHubba algorithms. (A) Degree, (B) MCC, (C) EPC, (D) DMNC.

Figure 5A shows the IS PPI network graph with 115 nodes, 104 edges, and a significant *P < *1.0 *E-16* value. Figure 5B shows the MI PPI network graph with 43 nodes, 19 edges, and a significant *P < 1.02 E-05* value. Inputting the MCC, DMNC, Degree, and EPC algorithms into the cytoHubba plug-in in Cytoscape resulted in 19 central IS protein-encoding genes, namely *FCGR1A, CCR7, S100A12, CD163, MMP9, IL2RB, CLEC4D, IL7R, HP, BCL2A1, CLEC5A, GZMK, CEACAM8, CR1, PTGS2, FLT3LG, NCR3, APRT*, and *CD79A* (Fig. 6). Furthermore, 13 central MI protein-encoding genes were found, namely *SPI1, HCK, IRF8, MYD88, LILRB3, CD300LB, SLC11A1, PRAM1, TRIM25, IFNGR2, RNPEP, LTBR*, and *POU2F2* (Fig. 7).

### 3.4. Determination of TFs and miRNAs of DEGs

**Figure 8.**
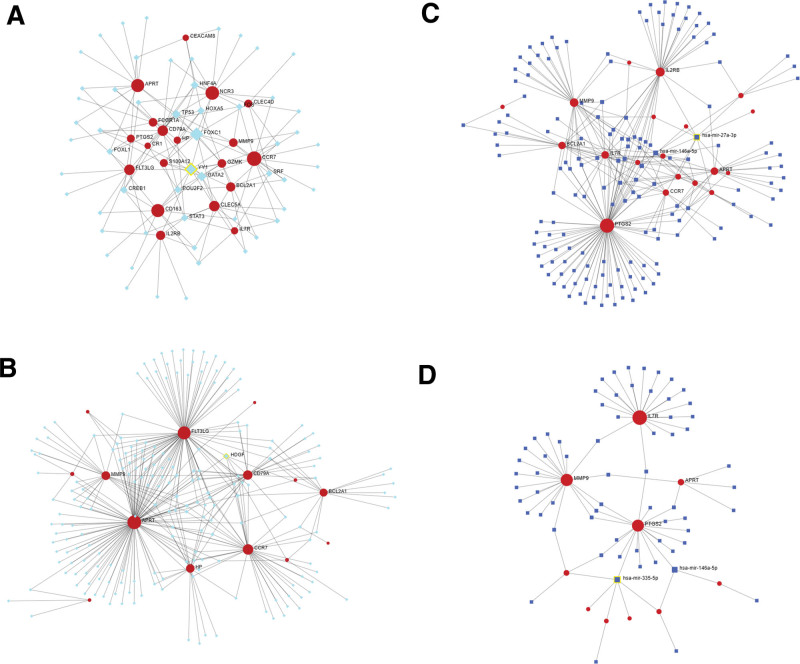
Visualization of ischemic stroke (IS) gene-transcription factor (TF) and microRNA (miRNA) interactions. (A) Network diagram showing IS differentially expressed genes (DEGs) and TFs from the JASPAR database. (B) Network diagram showing IS DEGs and TFs from the ENCODE database. (C) Network diagram showing DEG–miRNA interactions from the TarBase database. (D) Network diagram showing DEG–miRNA interactions from the miRTarBase database. Red circles represent genes, green squares represent TFs, and blue squares represent miRNAs. The size of the square indicates the strength of the relationship with the gene (the larger the square, the stronger the relationship and vice versa). ENCODE = Encyclopedia of DNA Elements.

Figure 8 shows the regulatory relationship of DEG-TFs and DEG-miRNAs in IS. Figure 8A shows that in the JASPAR database, the relationship between the *CCR7* gene and TFs in IS reached 14 degrees, and the correlation between the TFs FOXC1 and YY1 and genes reached 11 degrees. Figure 8B shows that in the ENCODE database, a 109-degree correlation between the *APRT* gene and TFs, and a 6-degree correlation between the gene and the TF HDGF were observed. Figure 8C shows the relationship between IS genes and miRNAs in the TarBase v8.0 database. The relationship between the *PTGS2* gene and miRNAs reached 85 degrees, whereas the relationship between the gene and hsa-miR-27a-3p reached 10 degrees. Figure 8D shows a 22-degree correlation between the *IL7R* gene and miRNA in the miRTarBase v8.0 database and a 6-degree correlation between hsa-miR-335-5p and gene.

**Figure 9.**
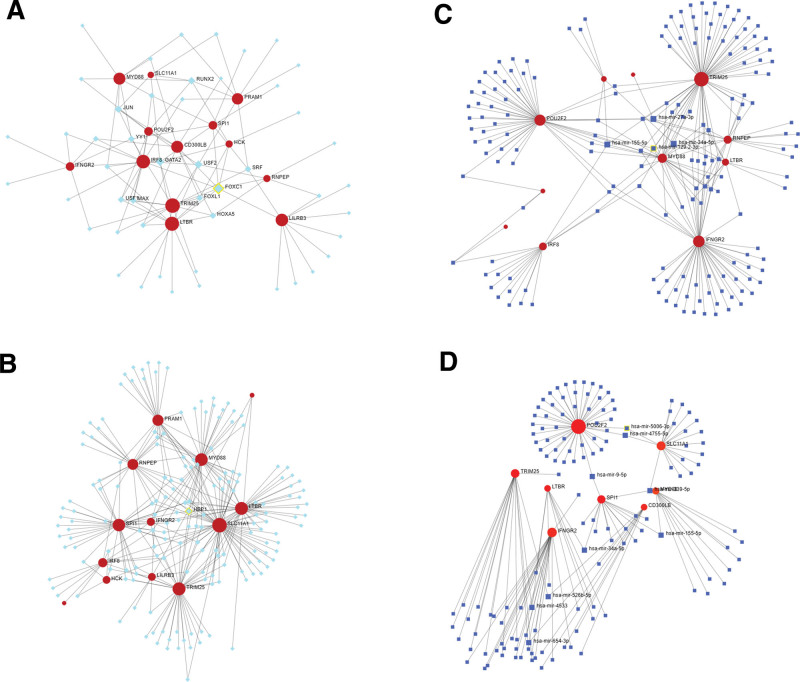
Myocardial infarction (MI) gene-transcription factor (TF) and -microRNA (miRNA) interactions. (A) Network diagram showing MI differentially expressed genes (DEGs) and TFs from the JASPAR database. (B) Network diagram showing MI DEGs and TFs from the ENCODE database. (C) Network diagram showing DEG–miRNA interactions from the TarBase database. (D) Network diagram showing DEG–miRNA interactions from the miRTarBase database. Red circles represent genes, green squares represent TFs, and blue squares represent miRNAs. The size of the square indicates the strength of the relationship with the gene (the larger the square, the stronger the relationship and vice versa). ENCODE = Encyclopedia of DNA Elements.

Figure 9A shows the relationship between MI genes and TFs in the JASPAR database. The relationship between the *TRIM25* gene and TFs reached 14 degrees, and the relationship between the TF FOXC1 and genes reached 8 degrees. Figure 9B shows the relationship between MI genes and TFs in the ENCODE database. The relationship between the *SLC11A1* gene and TFs reached 62 degrees, and the relationship between the TF HBP1 and genes reached 5 degrees. Figure 9C shows the relationship between MI genes and miRNAs in the TarBase v8.0 database. The relationship between *TRIM25* and miRNAs reached 65 degrees, and the relationship between hsa-miR-34a-5p, hsa-miR-155-5p, hsa-miR-27a-3p, and hsa-miR-129-2-3p and genes reached 5 degrees. Figure 9D shows the relationship between MI genes and miRNAs in the miRTarBase v8.0 database. The relationship between the *POU2F2* gene and miRNAs reached 49 degrees, and the relationship between hsa-miR-34a-5p, hsa-mir-9-5p, hsa-miR-155-5p, hsa-miR-339-5p, hsa-miR-526b-5p, hsa-miR-654-3p, hsa-miR-4533, hsa-miR-4755-5p, and hsa-miR-5006-3p and genes reached 2 degrees.

### 3.5. Predictive drug analysis

**Figure 10.**
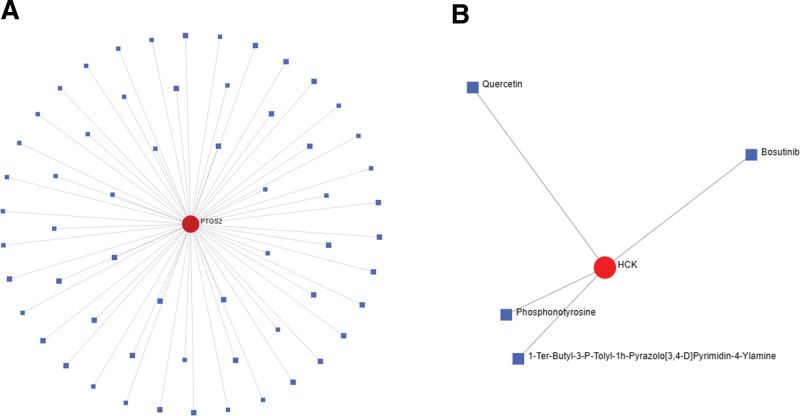
Drug–protein interactions. (A) Ischemic stroke. (B) Myocardial infarction.

A total of 19 central IS and 13 central MI proteins were used for drug prediction. The IS protein–drug interaction is shown in Figure 10A, where 30 nodes contain 1 gene (*PTGS2*) and 29 compounds. Five nodes for MI contained 1 gene (*HCK*) and 4 compounds, as shown in Figure 10B.

## 4. Discussion

In this study, 115 IS, 44 MI, and one common DEG were screened using the GEO database. GO functional analysis results showed that IS DEGs were involved in the regulation of biological processes, such as specific granule, specific granule membrane, and the immune response-regulating signaling pathway. MI DEGs were primarily involved in the response to type II interferon, myeloid leukocyte activation, and secretory granule membrane. KEGG enrichment analysis results showed that these DEGs were enriched in signaling pathways. IS DEGs were primarily enriched in transcriptional misregulation in cancer, *Salmonella* infection, hematopoietic cell lineage, the nuclear factor-kappa B (NF-κB) signaling pathway, viral protein interaction with cytokine and cytokine receptor, and the C-type lectin receptor signaling pathway. MI DEGs were primarily enriched in pathways, such as the NF-κB signaling pathway, osteoclast differentiation, and tuberculosis. The results of the PPI network and cytoHubba analyses showed a correlation between DEGs and 8 candidate genes with high confidence scores related to IS: *FCGR1A, CCR7, CLEC5A, GZMK, CD16*3*, MMP9, S100A12*, and *IL7R*. The candidate genes with high confidence scores associated with MI were *MYD88, SPI1, HCK, LILRB3, CD300LB, PRAM1, IRF8*, and *SLC11A1*. Via visualization of DEG-TFs, the TFs FOXC1, YY1, and HDGF were predicted to be important for IS, whereas via the visualization of DEG-miRNAs, hsa-miR-27a-3p and hsa-miR-335-5p were predicted to be crucial for IS. The TFs HBP1 and FOXC1 were important for MI, whereas visualization of DEG-miRNAs revealed hsa-miR-34a-5p, hsa-mir-9-5p, hsa-miR-155-5p, hsa-miR-339-5p, hsa-miR-526b-5p, hsa-miR-654-3p, hsa-miR-4533, hsa-miR-4755-5p, hsa-miR-5006-3p, hsa-miR-34a-5p, hsa-miR-155-5p, hsa-miR-27a-3p, and hsa-miR-129-2-3p to be important for MI. We also used DEG–drug predictive networks to identify drugs for IS and MI.

The pathophysiological mechanisms and common risk factors of cardiovascular and cerebrovascular diseases, including age, hypertension, and diabetes, are similar.^[13]^ However, there are still differences in the pathogenesis and molecular markers of the 2 diseases. The top 8 genes highly expressed in IS with high scores in 4 algorithms were selected. *FCGR1A* is an important gene in IS, and *CCR7* is a central chemokine receptor.^[14,15]^ Dendritic cell (DC) migration in the brain parenchyma depends on the chemokine receptor *CCR7*. DCs accumulate in the central nervous system during neuroinflammation, and the immune system plays a role in the pathology of IS.^[16]^ *IL7R* is a novel therapeutic target that promotes functional and histological recovery after IS.^[17]^ Following ischemic brain injury, the ensuing neuroinflammation can lead to additional damage and cell death.^[18]^ *CD163* is a monocyte/macrophage-specific marker primarily expressed on cells with strong anti-inflammatory potential. CD163-expressing mononuclear phagocytes, as well as soluble CD163, may be involved in the downregulation of inflammatory responses.^[19]^ *CD163* is also a potential inflammatory biomarker and therapeutic target.^[20]^ Thus, research on *CCR7* and *CD163* is of great importance in controlling inflammation after IS. *CLEC5A* is a C-type lectin domain family 5 member, which is related to the progression of various acute and chronic inflammatory diseases, and is primarily expressed in macrophages of atherosclerotic lesions.^[21]^ Its increased expression is associated with early plaque progression, promoting macrophage survival.^[22]^ Rupture of unstable atherosclerotic plaques is the pathological mechanism underlying acute ischemic events.^[23]^ High serum levels of MMP9 in the acute phase of IS are associated with an increased risk of death and severe disability. Therefore, serum MMP9 levels may be an important prognostic marker for IS.^[24]^

The top 8 genes highly expressed in MI with high scores in 4 algorithms were selected. *MYD88* is a key player in the inflammatory response, and evidence of pathological features indicates that it plays a role in cellular signaling pathways involved in the survival, proliferation, apoptosis, and autophagy of cardiomyocytes, endothelial cells, fibroblasts, monocytes, and stem cells during MI. Targeting these abnormal signaling pathways may help improve the pathological manifestations of MI.^[25]^ *MYD88* is of great value in reducing inflammation and restoring myocardial function after MI. The development of heart failure (HF) remains a common complication after AMI and is associated with severe adverse outcomes. *HCK* and *IRF8* were identified to be important in predicting the occurrence of HF after MI.^[26]^ Related studies have found that the upregulation of *SPI1* expression during MI can aggravate cardiac tissue damage and disease progression by activating the TLR4/NF-κB axis.^[27]^ Atherosclerosis is the main pathological basis of cardiovascular and cerebrovascular diseases.^[28]^ *LILRB3* is a reliable molecular biomarker for plaque status changes in stable coronary artery disease, ST-segment elevated MI progression, and MI recurrence.^[29]^

There also exist differences between the 2 diseases regarding pathways. IS pathways are primarily enriched in transcriptional misregulation in cancer, *Salmonella* infection, hematopoietic cell lineage, the NF-κB signaling pathway, viral protein interaction with cytokine and cytokine receptor, and the C-type lectin receptor signaling pathway. The NF-κB pathway is a typical proinflammatory signaling pathway, playing a crucial role in the functional outcome after stroke.^[30,31]^ In fact, in vitro studies have found that the Sirt1/NF-κB pathway substantially affects cerebral IS in rat neurons affected by apoptosis.^[32]^ Moreover, research in mice shows that inhibition of the NF-κB/NLRP3 signaling pathway partially reduced stroke-induced white matter damage and alleviated microglial pyroptosis.^[31]^ C-type lectin-1/Syk signaling plays a key role in inflammatory activation in IS, and the C-type lectin-1 antagonist LAM reduced infarct volume and improved neurological function 3 days after IS.^[33]^ MI DEGs were primarily enriched in pathways such as the NF-κB signaling pathway, osteoclast differentiation, and tuberculosis. Related studies have shown that osteoclast differentiation may play an important role in the acute phase of AMI.^[34]^

TFs and miRNAs regulate gene expression. The interaction between TF proteins and DNA underlies the regulation of transcription, a coordinated process that responds to environmental factors to achieve temporal and tissue specificity.^[35,36]^ The transcription of the identified TF FOXC1 in IS is induced by MDL-811 stimulation and is required for the anti-inflammatory effect of MDL-811. MDL-811 is also expressed in human monocytes isolated from IS patients. Studies have shown that neuroinflammation is considered a key factor in the progression of stroke and is of great importance during candidate drug screening for alternative treatments for IS.^[37]^ The HBP1 TF downregulates the expression of *HMGB1* and its related TLR4 and NF-κB, which has a protective effect against MI.^[38]^

miRNAs regulate gene expression by blocking mRNA translation and play an important role in many diseases.^[39]^ hsa-miR-27a-3p and hsa-miR-335-5p were crucial in IS, whereas hsa-miR-34a-5p was crucial in MI.^[40,41]^ The above information may reveal some unique mechanisms underlying the diseases, suggest new disease markers, and provide a possible basis for predicting IS and MI, as well as identify potential therapeutic targets. The prediction of drugs may have a role in disease prevention.

However, this study has certain limitations. The data were derived from public databases, which lacked clinical information for analysis. In addition, the limited sample size prevented the data from being divided into derivation and validation sets. Moreover, the lack of experimental research and validation is a shortcoming. Finally, no in vitro or in vivo assays were performed to validate the identified molecular biomarkers.

## 5. Conclusion

In this study, core DEGs related to IS and MI were detected using bioinformatics. KEGG pathways and GO terms were investigated. TFs and miRNAs important to core DEGs and gene–drug interrelationships were also analyzed. These core DEGs and regulatory molecules may be involved in the mechanisms underlying the development of IS and MI and thus may be used as potential diagnostic and therapeutic targets for these diseases.

## Author contributions

**Resources:** Guangming Wang.

**Software:** Min Wang, Yuan Gao.

**Visualization:** Huaqiu Chen, Ying Shen.

**Writing – original draft:** Min Wang, Yuan Gao.

**Writing – review & editing:** Jianjie Cheng, Guangming Wang.
